# Diagnostic Challenges of Methanol Poisoning in Resource‐Limited Settings: A Case Series

**DOI:** 10.1002/ccr3.73220

**Published:** 2026-07-30

**Authors:** Hongyun Chen, Liangbo Zeng, Baoliang Xiang, Ting Wu, Deju Li, Xianfeng Wu, Guanghua Pan

**Affiliations:** ^1^ Department of Emergency Medicine People's Hospital of Anshun Anshun Guizhou China; ^2^ Department of Emergency Medicine First Affiliated Hospital of Guangzhou Medical University Guangzhou Guangdong China

**Keywords:** methanol, misdiagnosis, poisoning

## Abstract

Methanol poisoning is a life‐threatening emergency, but diagnosis is challenging in resource‐limited settings lacking toxicology testing. This case series presents three cases of methanol poisoning: two patients with neurological symptoms and unexplained high anion gap metabolic acidosis were initially misdiagnosed as stroke or diabetic ketoacidosis and died after transfer, whereas the third patient received prompt empirical hemodialysis based on clinical suspicion and survived with full recovery. These cases illustrate that early recognition and empirical hemodialysis guided by clinical judgment, without confirmatory toxicology testing, can be life‐saving in resource‐constrained environments.

## Key Clinical Message

In resource‐limited settings lacking toxicology testing and specific antidotes, methanol poisoning should be suspected in patients with unexplained high anion gap metabolic acidosis, visual or neurological symptoms, and alcohol intake history. Neuroimaging provides additional diagnostic clues. Prompt empirical hemodialysis based on clinical suspicion can be life‐saving, as confirmed by this case series where delayed diagnosis led to fatalities.

## 1 Introduction

Methanol poisoning continues to be a significant public health challenge in certain regions, with outbreaks still reported in recent years [1, 2]. Exposure typically occurs through accidental ingestion of illicit alcohol containing toxic levels of methanol or via occupational contact. Methanol toxicity can lead to central nervous system depression, visual impairment, and high anion gap metabolic acidosis (HAGMA) [3]. In the early stages, poisoning presents with nonspecific mental status changes, and obtaining a reliable medical history is often difficult, making early identification a common challenge for emergency physicians.

Gas chromatography remains the gold standard for measuring methanol concentrations, yet it requires specialized laboratories and trained personnel. While point‐of‐care rapid testing methods such as formate assays [4] and portable breath detectors [5] have been explored, most remain in the prototype or limited rollout phase. Reliable methanol detection methods are still scarce, particularly in developing countries [1]. Without timely diagnosis and appropriate treatment, including antidotes and hemodialysis, the condition can deteriorate rapidly and prove fatal, especially following high‐dose exposure.

Methanol poisoning is relatively uncommon in China. Measurement of methanol levels is not a routine laboratory test in hospitals. In regions without access to toxicology testing centers, early identification relies on high clinical suspicion among frontline emergency physicians. The core diagnostic triad consists of severe unexplained HAGMA, neurological or visual symptoms, and a history of alcohol ingestion. This study reports three cases of acute methanol poisoning with different outcomes treated in our emergency department. The primary aim of this case series is not to discover novel clinical manifestations of methanol poisoning, but to translate established diagnostic principles into a practical bedside management framework for resource‐limited settings.

## 2 Case Presentation

### 2.1 Case 1

#### 2.1.1 Case History

A 62‐year‐old woman with a history of hypertension presented to the emergency department at 17:50 on December 2, 2024, reporting 1 day of dizziness and blurred vision, followed by lower back pain for 40 min.

#### 2.1.2 Examination

On presentation, her vital signs were as follows: temperature 36.2°C, blood pressure 133/69 mmHg, heart rate 50 beats per minute and regular, and respiratory rate 22 breaths per minute. She was conscious but lethargic. Pupils were equally dilated (5.0 mm) with sluggish light reflexes. Cardiac and pulmonary examinations were unremarkable. The neurological examination revealed no focal deficits. Initial arterial blood gas showed pH 6.83, PaO_2_ 167 mmHg, PaCO_2_ 12 mmHg, HCO_3_^−^ < 3.0 mmol/L, lactate 5.5 mmol/L, glucose 11.7 mmol/L, Na^+^ 136.4 mmol/L, Cl^−^ 105.6 mmol/L, K^+^ 4.47 mmol/L, and the calculated anion gap (AG) was 27.8 mmol/L (based on HCO_3_^−^ = 3.0 mmol/L). A cerebral CT scan demonstrated white matter demyelinating changes.

#### 2.1.3 Differential Diagnosis, Investigations, and Treatment

Upon presentation, the patient's symptoms of dizziness, blurred vision, and lethargy raised suspicion for acute cerebrovascular accident. Brain MRI revealed symmetrical abnormal signals in the bilateral frontal, temporal, parietal, and occipital lobes, as well as the basal ganglia (Figure 1A,B). On DWI, these lesions showed hyperintensity, with corresponding hypointensity on ADC mapping. The radiology report described these findings as “consistent with metabolic or toxic encephalopathy.” No direct communication between the radiology and clinical teams occurred regarding specific toxic etiologies. Consequently, a definitive diagnosis was not established. Supportive care including oxygen therapy, sodium bicarbonate infusion, analgesics, and fluid resuscitation was administered. Despite these interventions, the patient's condition deteriorated with no improvement in acidosis and persistent unresponsiveness. Per family request, she was transferred to another hospital that evening for further evaluation.

**FIGURE 1:**
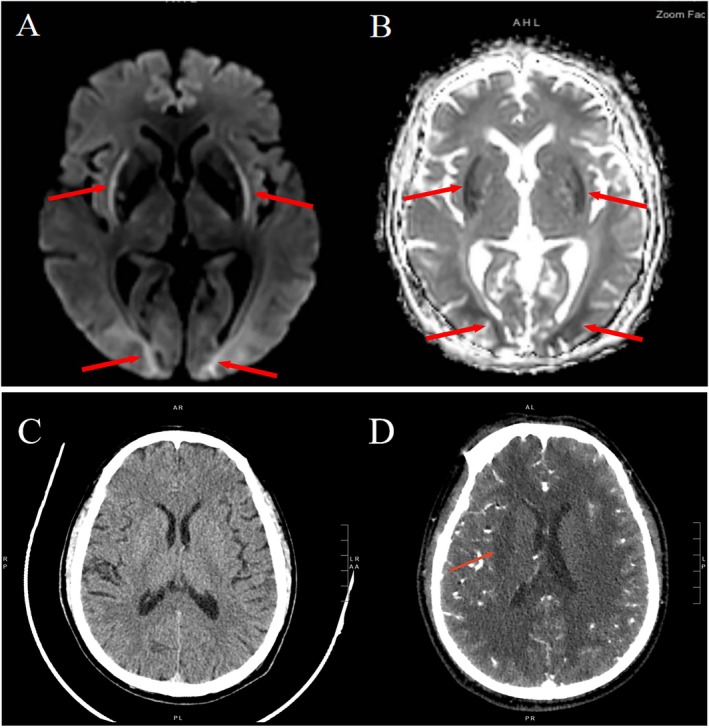
(A, B) Brain MRI scan of Case 1. (A) Diffusion‐weighted imaging (DWI) shows symmetric patchy hyperintense signals involving the bilateral putamen and occipital lobes. (B) The corresponding ADC map demonstrates reduced signal intensity in the same regions. *Clinical significance*: In a patient with altered mental status/visual symptoms and unexplained high anion gap metabolic acidosis, this specific MRI pattern strongly suggests methanol poisoning while confirmatory toxicology testing is pending. It does not replace laboratory confirmation but serves as a valuable diagnostic clue in resource‐limited settings where methanol levels are unavailable. (C, D) Non‐contrast brain CT and CT angiography in Case 2. (C) Non‐contrast brain CT shows no remarkable abnormality. (D) CT angiography shows decreased attenuation in the bilateral fronto‐temporo‐parieto‐occipital lobes, basal ganglia, and corpus callosum, with the red arrow marking the right basal ganglion.

#### 2.1.4 Outcome

The patient died despite intensive care after transfer.

#### 2.1.5 Follow‐Up

On December 5, 2024, investigators from the local Centers for Disease Control and Prevention (CDC) identified the patient as part of a methanol poisoning outbreak. The cluster was traced to a banquet at a rural restaurant, where 10 individuals had consumed contaminated alcohol. Eight attendees developed mild symptoms, while the two most severe cases (Case 1 and Case 2) presented to our emergency department and subsequently died. Laboratory analysis of the shared beverage confirmed methanol concentrations well above safety limits.

### 2.2 Case 2

#### 2.2.1 Case History

A 59‐year‐old man with hypertension and diabetes was admitted to the emergency department at 19:45 on December 3, 2024. The patient complained of dizziness and blurred vision for 1 day. He had a long‐standing history of heavy alcohol consumption and acknowledged continuous drinking during the 3 days prior to symptom onset.

#### 2.2.2 Examination

Physical examination revealed a temperature of 36.8°C, pulse 78 beats per minute, respiratory rate 20 breaths per minute, and blood pressure 135/80 mmHg. The patient was alert and oriented. Breath sounds were clear bilaterally on auscultation. Heart sounds were regular at 78 beats per minute without audible murmurs. No other positive findings were noted. Initial laboratory tests showed: pH 6.8, HCO_3_^−^ < 3.0 mmol/L, lactate 10.3 mmol/L, glucose 27.7 mmol/L, β‐hydroxybutyrate 1.24 mmol/L, Na^+^ 136.3 mmol/L, Cl^−^ 100.4 mmol/L, K^+^ 4.25 mmol/L, and the calculated AG was 32.9 mmol/L (based on HCO_3_^−^ = 3.0 mmol/L). Brain CT angiography demonstrated symmetrical injury involving bilateral cortical regions and basal ganglia, consistent with either metabolic encephalopathy or toxic brain injury (Figure 1C,D).

#### 2.2.3 Differential Diagnosis, Investigations, and Treatment

Given the patient's history of chronic heavy alcohol consumption and the presence of altered mental status, Wernicke encephalopathy was considered in the differential diagnosis. However, the classic triad (ophthalmoplegia, ataxia, confusion) was not fully present. Furthermore, Wernicke encephalopathy alone could not account for the profound metabolic acidosis observed in this patient. The presence of hyperglycemia and ketosis initially pointed to diabetic ketoacidosis (DKA), and treatment with insulin infusion and bicarbonate therapy was initiated. Despite these interventions, his neurological status worsened overnight, with progressive confusion and intractable seizures. A definitive diagnosis could not be established. Upon his family's insistence, he was transferred to a tertiary center on the afternoon of December 4 for further evaluation and management.

#### 2.2.4 Outcome

The patient died despite intensive care after transfer.

#### 2.2.5 Follow‐Up

The local CDC identified the patient as part of a methanol poisoning outbreak. Eight attendees developed mild symptoms, while the two most severe cases (Case 1 and Case 2) presented to our emergency department and subsequently died. Laboratory analysis of the shared beverage confirmed methanol concentrations well above safety limits.

### 2.3 Case 3

#### 2.3.1 Case History

A 53‐year‐old man arrived at the emergency department at 19:14 on October 17, 2025. The patient was found by his family in a state of confusion, with slurred speech, urinary and fecal incontinence, and dyspnea for 5 h after ingesting alcohol. He complained of nausea without vomiting, fever, hematemesis, or melena. His medical history included impaired liver function and pancreatitis.

#### 2.3.2 Examination

Physical examination revealed a temperature of 36.8°C, pulse 118 bpm, respiratory rate 24 bpm, and blood pressure 84/50 mmHg. The patient was delirious and uncooperative. Both pupils were equal, round, and 4 mm in diameter, with brisk light reflex. No jaundice was noted. Lung auscultation revealed no dry or moist rales. The abdomen was soft without tenderness or rebound tenderness. Moderate edema was present in both lower limbs. Initial arterial blood gas analysis showed pH < 6.8, PCO_2_ 11 mmHg, PO_2_ 137 mmHg, HCO_3_^−^ 6.5 mmol/L, Na^+^ 147.0 mmol/L, K^+^ 4.1 mmol/L, Cl^−^ 99.9 mmol/L, lactate > 15.0 mmol/L, AG 40.6 mmol/L, and blood glucose 2.8 mmol/L. Other laboratory results included: total bilirubin 57.1 μmol/L, direct bilirubin 19.7 μmol/L, alanine aminotransferase (ALT) 109.7 U/L, creatinine 244.9 μmol/L, amylase 516.4 U/L, and lipase 104.8 U/L. Brain CT demonstrated white matter demyelination.

#### 2.3.3 Differential Diagnosis, Investigations, and Treatment

Based on the history of alcohol consumption, acute altered mental status, and severe HAGMA, and drawing lessons from Case 1 and Case 2, acute methanol poisoning was strongly suspected. A blood sample was sent to an out‐of‐province reference laboratory for definitive methanol concentration analysis. Fomepizole, the preferred antidote, was not available at our hospital. Ethanol was considered inappropriate for empirical use without confirmatory toxicology results. Therefore, empirical intermittent hemodialysis was initiated immediately without waiting for toxicology results and was continued for 2 h. Post‐dialysis blood gas showed significant improvement, with pH 7.49, PCO_2_ 25 mmHg, HCO_3_^−^ 19.1 mmol/L, Na^+^ 144.0 mmol/L, K^+^ 3.2 mmol/L, Cl^−^ 97.9 mmol/L, lactate 9.8 mmol/L, and the calculated post‐dialysis AG was 27.0 mmol/L. The patient regained consciousness and his condition gradually improved. Follow‐up testing 1 week after admission revealed a serum methanol concentration of 11.71 mg/L.

#### 2.3.4 Outcome

The patient remained clinically stable and was discharged after 10 days with significant improvement.

#### 2.3.5 Follow‐Up

The patient recovered fully and had quit drinking at the 3‐month follow‐up.

To identify key differences between the fatal outcomes in Case 1 and Case 2 and the survival of Case 3, we compared their clinical presentations, management, and outcomes in Table 1.

**TABLE 1:** Comparison of clinical features, interventions, and outcomes among three methanol poisoning cases.

Parameter	Case 1	Case 2	Case 3
Age/sex	62/F	59/M	53/M
Time to presentation	02‐12‐2024 17:50	03‐12‐2024 19:45	17‐10‐2025 19:14
Alcohol history	Unclear	Clear history before onset	Clear history before onset
Chief symptoms	Dizziness, blurred vision	Dizziness, blurred vision	Confusion, slurred speech, incontinence, dyspnea
Initial suspected diagnosis	Cerebrovascular accident	DKA, Wernicke encephalopathy	Methanol poisoning
pH/HCO_3_^−^ (mmol/L)/AG (mmol/L)	6.83/< 3.0/27.8	6.8/< 3.0/32.9	< 6.8/6.5/40.6
Glucose (mmol/L)	11.7	27.7	2.8
Lactate (mmol/L)	5.5	10.3	> 15
Imaging findings	MRI: symmetrical bilateral occipital & basal ganglia lesions	CT: symmetrical bilateral cortical & basal ganglia injury	CT: white matter demyelination
Methanol poisoning suspected at presentation	No	No	Yes (high clinical suspicion)
Fomepizole/ethanol administered	No	No	No
Hemodialysis performed	No	No	Empirical (on admission day)
Outcome	Died	Died	Survived

Abbreviations: AG, anion gap; CT, computed tomography; DKA, diabetic ketoacidosis; MRI, magnetic resonance imaging.

## 3 Discussion

This report presents three methanol poisoning cases managed in a resource‐limited setting. Two patients died due to delayed diagnosis while the third survived after urgent empirical hemodialysis. The key finding is that methanol poisoning is diagnostically challenging without access to rapid toxicological testing; however, timely empirical treatment based on clinical suspicion can significantly improve outcomes.

The typical symptoms of methanol poisoning include loss of consciousness, visual impairment including blurred vision or blindness, and severe metabolic acidosis. However, interindividual variability exists in clinical practice. The differences in ADH genotypes may have influenced the rate at which methanol is metabolized into formic acid, leading to variations in clinical manifestations [6]. Acute pancreatitis manifested by abdominal pain has also been reported in cases of methanol poisoning [7]. Moreover, Case 2 shows that methanol poisoning and DKA can coexist, which further complicates diagnosis. This result is supported by the findings of Erfanifar et al., who first reported this overlap in 2022 [8]. Both conditions result in HAGMA, potentially leading to the attribution of severe acidosis solely to DKA. Previous studies have also shown that the presence of hyperglycemia is closely associated with increased mortality in methanol poisoning patients [9]. Furthermore, methanol poisoning induces pancreatic injury. Thus, patients with a history of diabetes who develop hyperglycemia subsequent to methanol poisoning are at a higher risk of developing DKA. In the absence of methanol/formate testing, severe HAGMA is extremely likely to be solely attributed to DKA, which delays the administration of antidote therapy. In the present case, the patient's DKA symptoms initially diverted clinicians' attention away from methanol poisoning, highlighting the need for clinicians to consider toxic causes when DKA is unusually severe or fails to respond to standard treatment.

In retrospect, the osmolar gap (OG) was not calculated in the initial workup of our cases, as our point‐of‐care testing (POCT) platform did not include serum osmolality measurement. In methanol poisoning, an elevated OG (typically > 10 mOsm/kg) provides an early, rapid bedside clue before acidosis fully develops, reflecting the presence of unmetabolized methanol. While OG has limitations in late‐presenting patients after methanol is metabolized to formic acid, its calculation would have strengthened the diagnostic suspicion early in the disease course. Recent evidence shows that admission lactate, lactate clearance, anion gap, and base excess are strong prognostic indicators in methanol poisoning [10]. Additionally, biochemical markers such as albumin‐corrected anion gap (ACAG) and lactate‐to‐albumin ratio (LAR) are strong predictors of prognosis in methanol poisoning, highlighting the importance of early recognition of severe metabolic derangements in guiding timely management decisions [11].

In cases where patient history is unclear, and detection methods are absent, typical neuroimaging can provide valuable diagnostic clues. MRI is well established for diagnosing and prognosticating methanol poisoning, and a meta‐analysis of 15 studies found that putaminal necrosis occurred in 47.6% of cases [12]. Diffusion‐weighted imaging (DWI) with apparent diffusion coefficient (ADC) mapping can distinguish reversible vasogenic edema (normal or increased ADC) from irreversible cytotoxic edema (restricted diffusion, low ADC), the latter predicting poor neurological outcome [13]. The MRI findings in Figure 1 indicated cytotoxic edema. In retrospect, it should have immediately raised the specific suspicion of methanol poisoning. However, this diagnostic opportunity was missed due to poor communication between the clinical and radiology teams. The radiologist, provided with limited clinical information, appropriately described the findings as “metabolic or toxic encephalopathy” without specifying methanol poisoning. The clinical team, in turn, did not integrate the imaging findings with the patient's HAGMA, altered mental status, and visual symptoms. Based on this experience, we propose that in resource‐limited settings without toxicology testing, routine reporting protocols for toxic encephalopathy should prompt radiologists to explicitly mention methanol poisoning when bilateral putaminal involvement with restricted diffusion is seen, particularly when clinical information includes HAGMA. Conversely, such MRI findings should prompt clinicians to include methanol poisoning in the differential diagnosis and support the decision to initiate empirical hemodialysis while confirmatory laboratory testing is pending. Therefore, early MRI should be strongly considered when initial CT is unrevealing.

Beyond clinical and imaging challenges, diagnostic delays in methanol poisoning are frequently exacerbated by specific, identifiable cognitive biases and systemic gaps. In resource‐limited settings where toxicological testing is unavailable, clinicians may develop a persistently low index of suspicion specifically for methanol poisoning, a form of availability bias. This systemic absence not only limits diagnostic confirmation but also reinforces a practice pattern in which methanol poisoning is routinely excluded from differential lists. In the first two cases presented here, anchoring bias, the most common cognitive bias in emergency medicine [14], led physicians to prioritize more common conditions (e.g., stroke, DKA) despite the presence of HAGMA and altered mental status. Confirmation bias further delayed diagnosis, as early clinical findings were interpreted as supportive of initial hypotheses rather than prompting reconsideration of toxic causes. Failure to recognize the temporal clustering of the two cases reflects weak cluster detection, which is a known contributor to delayed outbreak recognition. During the COVID‐19 pandemic, delayed identification of case clusters was associated with increased mortality [15]. In low‐resource emergency departments, awareness of and adherence to cluster case surveillance protocols remain low, likely exacerbating diagnostic delays. As a potential countermeasure to these cognitive and system‐level challenges, machine learning‐based clinical decision support integrated into electronic health records, similar to existing sepsis early warning systems [16], could potentially assist in the early recognition of methanol poisoning by continuously screening for patterns of neurological symptoms and unexplained HAGMA in resource‐limited settings.

Hemodialysis directly removes methanol and formate, and is considered the primary extracorporeal treatment for methanol poisoning, especially where antidotes (fomepizole) are unavailable [3]. The EXTRIP consensus guidelines provide evidence‐based indications for hemodialysis in methanol poisoning. Severe methanol poisoning, defined by coma, seizures, new vision deficits, metabolic acidosis with blood pH ≤ 7.15, persistent acidosis despite supportive care, or a serum anion gap > 24 mmol/L, is a strong indication for hemodialysis (Grade 1D) [17]. Applying these criteria to our three cases, all met the threshold for immediate hemodialysis at presentation. However, only Case 3 received empirical hemodialysis promptly upon clinical suspicion, without waiting for toxicology results. In contrast, Case 1 and Case 2 were transferred to another hospital without receiving hemodialysis at our facility, resulting in critical delays in definitive treatment. The stark difference in outcomes, death in Case 1 and Case 2 versus survival and full recovery in Case 3, underscores that timely initiation of hemodialysis based on clinical criteria, rather than confirmatory testing, is the key determinant of survival in resource‐limited settings where rapid toxicology is unavailable. However, timing alone does not fully explain the different outcomes. In Case 1 and Case 2, methanol poisoning was never considered in the differential diagnosis. Consequently, no diagnostic workup or hemodialysis was attempted before transfer. In contrast, by the time Case 3 presented, we had learned from the previous two fatalities. Methanol poisoning had been added to our differential diagnosis list for patients presenting with alcohol intake, altered mental status, and severe HAGMA. This change in clinical suspicion, rather than any difference in presenting severity, allowed us to initiate empirical hemodialysis immediately.

In our Case 3, the retrospectively measured serum methanol concentration was only 11.71 mg/L, far below any guideline‐recommended threshold [17]. Methanol itself is relatively nontoxic; its metabolites, particularly formic acid, cause the metabolic acidosis and end organ damage. The patient presented several hours after alcohol consumption, allowing most of the absorbed methanol to be metabolized to formate. By the time blood was drawn, the parent methanol concentration had fallen dramatically, whereas formate, which is not routinely measured, remained high and drove the severe acidosis. Thus, a low or even undetectable methanol concentration does not exclude methanol poisoning in a patient with otherwise typical features. In such situations, the anion gap, blood pH, and osmolal gap (if available) are more reliable indicators for initiating empirical hemodialysis.

Based on our experience with these three cases and the existing literature, we propose a practical clinical decision algorithm for emergency physicians managing suspected methanol poisoning in resource‐limited settings (Figure 2). To implement this algorithm, we mandate explicit consideration of methanol poisoning for any unexplained HAGMA, regardless of coexisting alternative diagnoses. We also require direct radiologist‐clinician communication when characteristic MRI findings are present. Finally, we adopt a low threshold for empirical hemodialysis based solely on clinical suspicion. Future integration of machine learning‐based clinical decision support into electronic health records may further aid early recognition of methanol poisoning in resource‐limited settings.

**FIGURE 2:**
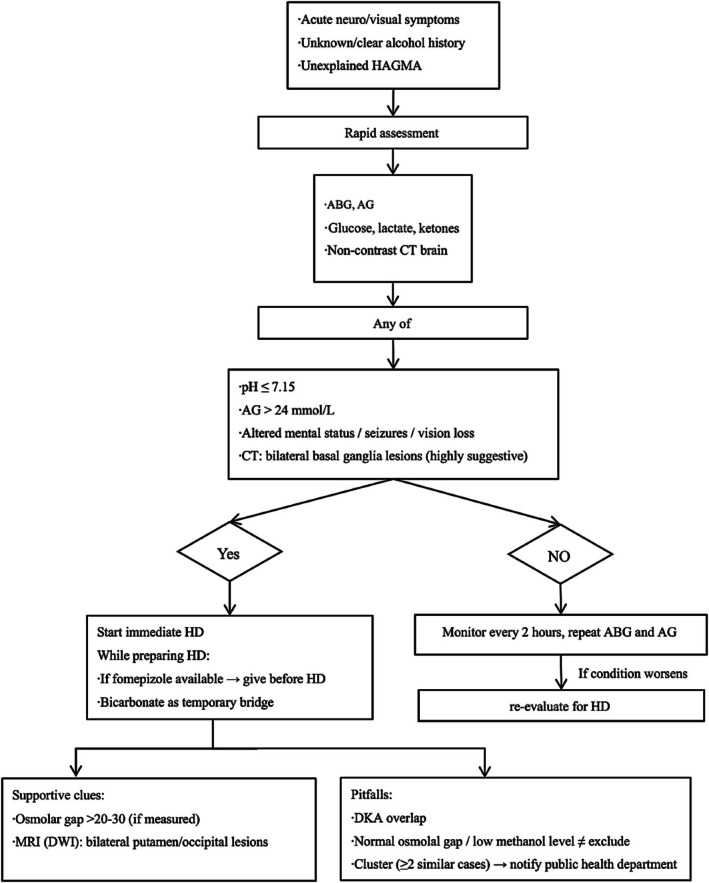
A practical clinical decision algorithm for the diagnosis and empirical management of suspected methanol poisoning in resource‐limited settings. Abbreviations: ABG, arterial blood gas; AG, anion gap; CT, computed tomography; DKA, diabetic ketoacidosis; DWI, diffusion‐weighted imaging; HAGMA, high anion gap metabolic acidosis; HD, hemodialysis; MRI, magnetic resonance imaging.

In resource‐limited settings without toxicological testing or methanol antidotes, this case series suggests that proactive clinical judgment, specifically considering methanol in unexplained HAGMA and initiating empirical hemodialysis, may improve outcomes. Neuroimaging and cluster exposure history may offer diagnostic clues. Systemic improvements like training and surveillance could address diagnostic gaps. Given the hypothesis‐generating nature of these findings, prospective validation is needed.

## Author Contributions

**Hongyun Chen:** data curation, investigation, resources, writing – original draft. **Liangbo Zeng:** conceptualization, methodology, supervision, writing – review and editing. **Baoliang Xiang:** data curation, writing – review and editing. **Ting Wu:** writing – review and editing. **Deju Li:** data curation, resources. **Xianfeng Wu:** writing – review and editing. **Guanghua Pan:** funding acquisition, writing – review and editing.

## Funding

This research was supported by the Anshun Science and Technology Project in Guizhou Province of China (nos. KeShe 2024‐69 and KeShe 2024‐41).

## Ethics Statement

This study was approved by the ethical committee of the People's Hospital of Anshun (no. 2025‐08).

## Consent

The authors declare that written informed consent has been obtained from all three patients for the publication of this manuscript and accompanying images. All identifying information, including names, dates of birth, and any facial or unique identifiable features in images has been excluded from the manuscript by the authors to prevent patient identification.

## Conflicts of Interest

The authors declare no conflicts of interest.

## Data Availability

The data are available with the corresponding author and can be reached on request.

## References

[1] M. Sadeghi , M. Fakhar , S. M. Hoseininejad , Z. Zakariaei , and A. Sadeghi , “The Clinico‐Epidemiological, Diagnostic and Therapeutic Aspects of Methanol Poisoning: A Five‐Year Retrospective Study, Northern Iran,” Drug and Alcohol Dependence 253 (2023): 111024. 38006673 10.1016/j.drugalcdep.2023.111024

[2] F. Alhusain , M. Alshalhoub , M. B. Homaid , L. C. A. Esba , M. Alghafees , and M. Al Deeb , “Clinical Presentation and Management of Methanol Poisoning Outbreaks in Riyadh, Saudi Arabia: A Retrospective Analysis,” BMC Emergency Medicine 24 (2024): 64. 38627622 10.1186/s12873-024-00976-1 PMC11020920

[3] Z. Nekoukar , Z. Zakariaei , F. Taghizadeh , et al., “Methanol Poisoning as a New World Challenge: A Review,” Annals of Medicine and Surgery (London) 66 (2021): 102445. 10.1016/j.amsu.2021.102445 PMC8187162 34141419

[4] K. E. Hovda , Y. E. Lao , G. Gadeholt , and D. Jacobsen , “Formate Test for Bedside Diagnosis of Methanol Poisoning,” Basic & Clinical Pharmacology & Toxicology 129, no. 1 (2021): 86–88. 33915025 10.1111/bcpt.13597

[5] J. van den Broek , D. Bischof , N. Derron , et al., “Screening Methanol Poisoning With a Portable Breath Detector,” Analytical Chemistry 93, no. 2 (2020): 1170–1178. 33315383 10.1021/acs.analchem.0c04230

[6] M. Tian , H. He , Y. Liu , et al., “Fatal Methanol Poisoning With Different Clinical and Autopsy Findings: Case Report and Literature Review,” Legal Medicine (Tokyo, Japan) 54 (2021): 101995. 34844153 10.1016/j.legalmed.2021.101995

[7] M. Sadeghi , Z. Zakariaei , M. Fakhar , R. Tabaripour , E. S. Banimostafavi , and H. Azadeh , “Acute Pancreatitis due to Methanol Toxicity During the COVID‐19 Pandemic,” Clinical Case Reports 9, no. 10 (2021): e04943. 34667611 10.1002/ccr3.4943 PMC8511877

[8] A. Erfanifar , M. Mahjani , S. Salimpour , N. Zamani , and H. Hassanian‐Moghaddam , “Diabetic Ketoacidosis as a Complication of Methanol Poisoning; A Case Report,” BMC Endocrine Disorders 22 (2022): 148. 35655196 10.1186/s12902-022-01037-z PMC9164356

[9] H. Sanaei‐Zadeh , S. K. Esfeh , N. Zamani , S. Kazemi Esfeh , F. Jamshidi , and S. Shadnia , “Hyperglycemia Is a Strong Prognostic Factor of Lethality in Methanol Poisoning,” Journal of Medical Toxicology 7, no. 3 (2011): 189–194. 21336799 10.1007/s13181-011-0142-x PMC3550199

[10] E. Ermete Güler , E. S. Bora , M. G. Efgan , and S. Bilgin , “Beyond Lactate: Prognostic Value of Metabolic and Biochemical Parameters in Methanol Poisoning‐A Retrospective Observational Study,” BMC Emergency Medicine 6, no. 1 (2026): 71. 10.1186/s12873-026-01492-0 PMC12977729 41654740

[11] İ. Ağaçkıran and M. Ağaçkıran , “Lactate‐to‐Albumin Ratio and Albumin‐Corrected Anion Gap as Predictors of Outcome in Methanol Poisoning: A Retrospective Observational Study,” PLoS One 20, no. 12 (2025): e0336382. 41343570 10.1371/journal.pone.0336382 PMC12677794

[12] R. O. da Silva , V. A. Ohannesian , B. N. Aguirre , et al., “Cranial Magnetic Resonance Imaging Findings in Methanol Poisoning: Systematic Review and Meta‐Analysis of Radiological Prevalence,” Neuroradiology Journal (2026): 19714009261423701, 10.1177/19714009261423701. 41711403 PMC12920168

[13] Y. Yang and H. Zhao , “Diffusion‐Weighted Imaging in Methanol Poisoning: Beyond the Lentiform Fork Sign,” Clinical Toxicology (Philadelphia, PA) 62, no. 1 (2024): 61–63. 38231203 10.1080/15563650.2024.2302452

[14] T. Watari , Y. Tokuda , Y. Amano , K. Onigata , and H. Kanda , “Cognitive Bias and Diagnostic Errors Among Physicians in Japan: A Self‐Reflection Survey,” International Journal of Environmental Research and Public Health 19, no. 8 (2022): 4645. 35457511 10.3390/ijerph19084645 PMC9032995

[15] R. S. Mousavi‐Roknabadi , M. Arzhangzadeh , H. Safaei‐Firouzabadi , et al., “Methanol Poisoning During COVID‐19 Pandemic: A Systematic Scoping Review,” American Journal of Emergency Medicine 52 (2021): 69–84. 34883289 10.1016/j.ajem.2021.11.026 PMC8611855

[16] N. Kijpaisalratana , J. Saoraya , P. Nhuboonkaew , et al., “Real‐Time Machine Learning‐Assisted Sepsis Alert Enhances the Timeliness of Antibiotic Administration and Diagnostic Accuracy in Emergency Department Patients With Sepsis: A Cluster‐Randomized Trial,” Critical Care 28, no. 1 (2024): 49. 38381351 10.1007/s11739-024-03535-5

[17] D. M. Roberts , C. Yates , B. Megarbane , et al., “Recommendations for the Role of Extracorporeal Treatments in the Management of Acute Methanol Poisoning: A Systematic Review and Consensus Statement,” Critical Care Medicine 43, no. 2 (2015): 461–472. 25493973 10.1097/CCM.0000000000000708

